# The L‐type calcium channel CaV1.3: A potential target for cancer therapy

**DOI:** 10.1111/jcmm.70123

**Published:** 2024-10-04

**Authors:** Xuerun Liu, Boqiang Shen, Jingyi Zhou, Juan Hao, Jianliu Wang

**Affiliations:** ^1^ Department of Gynecology and Obstetrics Peking University People's Hospital Beijing China

**Keywords:** CACNA1D, calcium channel blockers, cancer, L‐type calcium channel, targeted therapy

## Abstract

Cancer remains a prominent cause to life expectancy, and targeted cancer therapy stands as a pivotal approach in contemporary therapy. Calcium (Ca^2+^) signalling plays a multifaceted role in cancer progression, such as proliferation, invasion and distant metastasis. Otherwise, it also exerts an important influence on the efficacy of clinical treatment, including cancer therapy resistance. In this review we discuss the role of the L‐type calcium channel CaV1.3 (calcium voltage‐gated channel subunit alpha1 D) in different types of cancers, highlighting its potential as a therapeutic target for certain cancer types. The development of selective blockers of the CaV1.3 channel has been of great interest and is expected to be a new option for the treatment of cancers such as prostate cancer and endometrial cancer. We present the pharmacological properties of CaV1.3 and the current status of selective blocker development, and analyse the challenges and possible directions for breakthroughs in the development of tailored medicines.

## 1 INTRODUCTION

Globally, cancer is the leading cause of death and a major obstacle to increasing life expectancy. According to World Health Organization statistics, cancer is the first or second leading cause of death before age 70 in 112 countries worldwide. In 2020, there were about 19.3 million new cancer cases and nearly 10 million cancer deaths worldwide.^1^ Female breast cancer has surpassed lung cancer as the most commonly diagnosed cancer, with an estimated 2.3 million new cases (11.7%), but lung cancer remains the leading cause of cancer deaths, with an estimated 1.8 million deaths (18%).^1^ Among men, lung cancer is the most common cancer and the leading cause of cancer deaths. Liver cancer and colorectal cancer are the second and third leading causes of cancer deaths in men, respectively. For women, breast cancer is the most common cancer and the leading cause of cancer deaths, followed by colorectal and lung cancer for incidence, and vice versa for mortality.^1^ The economic costs of cancer globally are enormous. It is estimated that from 2020 to 2050, the global economic cost of cancer will be approximately $25.2 trillion, equivalent to a 0.55% tax annually.^2^ With the continuous deepening of research, people have a clearer and clearer understanding of the mechanism of tumorigenesis, and new methods and drugs for the treatment of cancers are emerging, and the cure rate and survival time of cancer patients have been greatly improved. Although a massive amount of work has been done in this area, there is still a long way to go to conquer cancers.

Voltage‐gated calcium channels (VGCCs) are key transducers of membrane potential changes into intracellular calcium transients that initiate many physiological events. Members of different VGCC families play different roles in cellular signalling. The CaV1 subfamily initiates contraction, secretion, regulation of gene expression, integration of synaptic inputs in neurons and synaptic transmission at ribbon synapses in specialized sensory cells.^3^ The CaV2 subfamily is primarily responsible for initiating synaptic transmission at fast synapses.^3^ The CaV3 subfamily plays an important role in the repetitive firing of action potentials in cardiac myocytes and thalamic neurons, among other rhythmically firing cells.^3^ CaV1.3 is a member of the L‐type VGCC family, and in recent years, more and more studies have shown that CaV1.3 is associated with cancer development, and CaV1.3 is a potential therapeutic target and prognostic biomarker for cancer therapy. The development of CaV1.3 selective blockers can not only be used as an experimental tool to study CaV1.3 in greater depth, but also is expected to become a new drug for cancer therapy. The high amino acid sequence similarity between CaV1.3 and CaV1.2 makes specific blocker development challenging. Although the discovery of CaV1.3 selective blockers has been reported in many literatures, their selectivity remains controversial.^4^

In this review, we provide an overview of the structure, physiology, and pathophysiology of the CaV1.3, with a focus on the role of CaV1.3 in cancers and the mechanisms of its regulation. In addition, we analyse the pharmacology of CaV1.3 and focus on the current status of CaV1.3 selective inhibitor development. We hope that this review will increase the understanding of CaV1.3 in cancers and provide new possibilities for personalized treatment of cancers.

## 2 STRUCTURE AND PATHOPHYSIOLOGY OF CaV1.3

**FIGURE 1:**
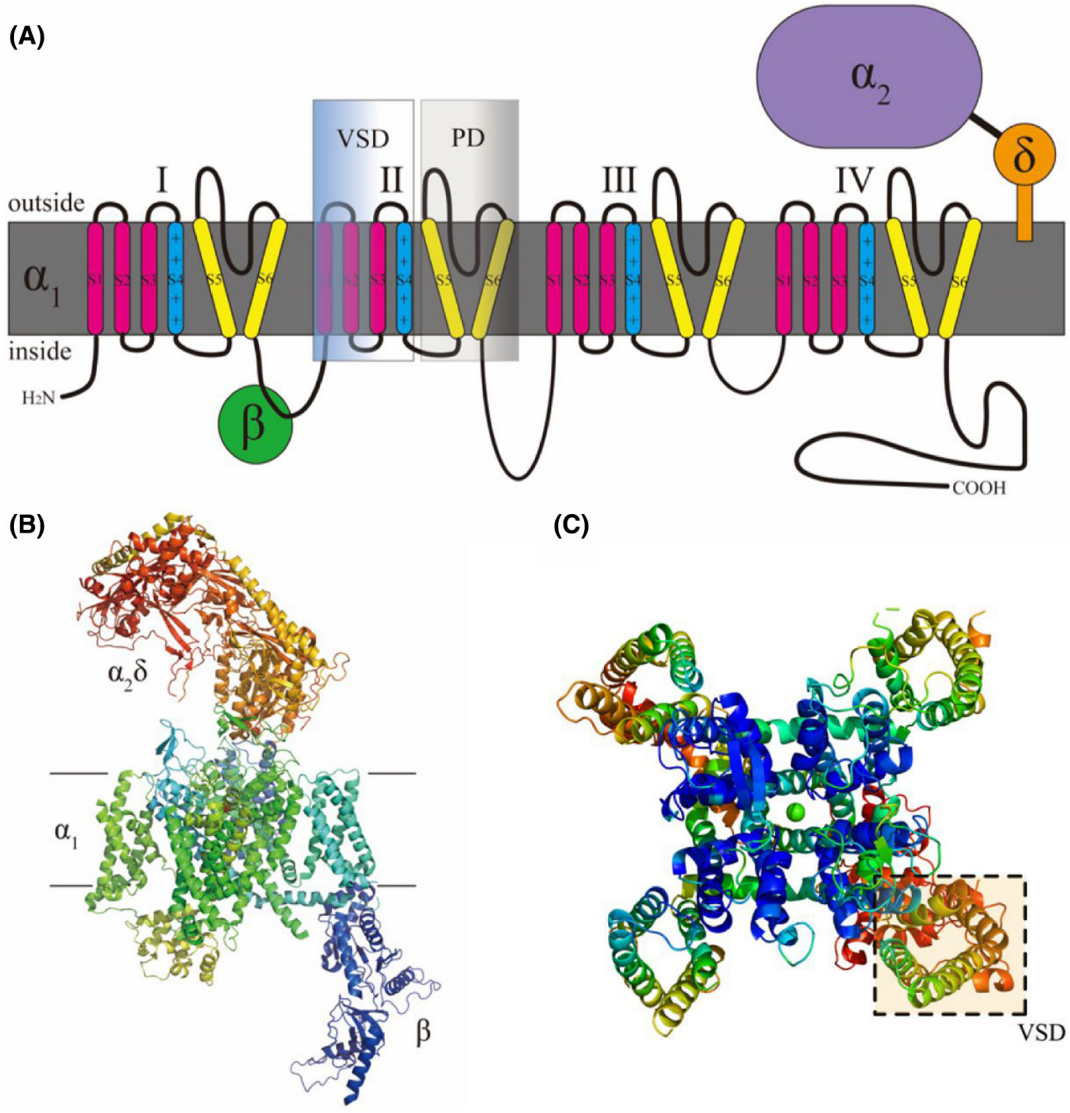
Schematic structure of CaV1.3. (A) CaV1.3 subunit topology map. The CaV1.3 α1 subunit contains four homologous structural domains (I–IV), each with six transmembrane fragments (S1–S6). S1–S4 constitute the voltage‐sensing domains (VSD) and S5 and S6 constitute the pore domain (PD). (B) Structure of human CaV1.3 (Protein Data Bank: 7UHG) shown from the membrane. (C) Observation of the α1 subunit from the extracellular view shows a domain exchange structure.

More than 50 years ago, Bernard Katz and Ricardo Miledi demonstrated the importance of calcium ions as an essential link in the coupling process of axonal terminal ‘electro‐secretory’ in their studies of squid giant synapses.^5^ VGCCs represent a critical link between electrical signals and non‐electrical processes such as muscle contraction, secretion and transcription. VGCC is a multiprotein aggregate that consists of a 190KD transmembrane α1 subunit, a 170KD dimeric structure consisting of a disulfide bond connecting α2 and δ, a 55KD intracellularly phosphorylated β‐subunit and a 33KD transmembrane γ‐subunit.^6^ The biological function of VGCCs is mainly determined by the voltage‐sensitive α1 subunit. VGCCs can be categorized into L‐type, T‐type, N‐type, P/Q‐type, and R‐type calcium channels according to the type of α1 subunit. The CaV α1 subunit consists of four repetitive structural domains (I–IV), each containing six transmembrane fragments (S1–S6) (Figure 1A).^3^ The S1 to S4 α‐helices contribute to voltage sensing, and the S5 and S6 fragments, together with the reentrant P‐loops of each structural domain, constitute transmembrane pores for Ca^2+^ entry and exit (Figure 1B,C).^7^ VGCCs can be subdivided into two main categories, low‐VGCCs and high‐VGCCs, depending on the activation threshold. Low‐VGCCs are mainly T‐type calcium channels, which are activated at about −60mv. High‐VGCCs consist of L‐type calcium channels (LTCC), P/Q‐type calcium channels, N‐type calcium channels and R‐type calcium channels, which are activated at about −20mv. They play different roles in cell signalling, and small changes in VGCCs can lead to human channelopathies (Table 1).^8^

**TABLE 1:** Classification, function and inhibitors of voltage‐gated calcium channels.

CAPS	Current	Gene	Protein	Main distribution	Channelopathy	Specific Antagonist
HVA	L	CACNA1S	CaV1.1	Skeletal muscle	Malignant hypothermia type 5, hypokalemic periodic paralysis type 1^8^	Dihydropyridines, phenylalkylamines, benzothiazepines
	L	CACNA1C	CaV1.2	Brain, pancreatic islets, adrenal, intestinal, bladder smooth muscle, vascular system, heart	Timothy syndrome,^8^ brugada syndrome type 3	Dihydropyridines, phenylalkylamines, benzothiazepines
	L	CACNA1D	CaV1.3	Brain, auditory hair cells, vestibular hair cells, retina,^9^ pancreatic islets, adrenal, kidney,^10^ heart	Sinoatrial node dysfunction and deafness syndrome, primary aldosteronism,^11^ neurodevelopmental syndrome with or without endocrine symptoms,^11^ autism spectrum disorder^12^	Dihydropyridines, phenylalkylamines, benzothiazepines
	L	CACNA1F	CaV1.4	Retina, T lymphocytes^13^	Congenital stationary night blindness type 2,^8^ x‐linked cone‐rod dystrophy type 3^14^	Dihydropyridines, Phenylalkylamines, Benzothiazepines
	P/Q	CACNA1A	CaV2.1	Central synapses, neuromuscular junction	Spinocerebellar ataxia type 6,^14^ episodic ataxia type 2,^14^ familial hemiplegic migraine type 1, congenital ataxia, developmental epileptic encephalopathy	ω‐Agatoxin IVA^15^
	N	CACNA1B	CaV2.2	Central and peripheral synapses	Myoclonus‐dystonia syndrome	Ziconotide,^16^ ω‐Conotoxin^17^
	R	CACNA1E	CaV2.3	Central and peripheral synapses	Developmental epileptic encephalopathy^14^	SNX‐482^18^
LVA	T	CACNA1G	CaV3.1	Neurons, cardiac muscle, smooth muscle	Childhood cerebellar atrophy, autosomal dominant cerebellar ataxia, juvenile myoclonus epilepsy	ProTx‐I^19^
	T	CACNA1H	CaV3.2	Neurons, Cardiac muscle, Smooth muscle	Childhood absence epilepsy,^14^ autism spectrum disorder,^14^ primary aldosteronism^14^	MCP‐1,^20^ N‐[[1‐[2‐(tert‐butylcarbamoylamino) ethyl]‐4‐(hydroxymethyl)‐4piperidyl] methyl]‐3, 5‐dichloro‐benzamide,^21^ N‐(1‐adamantyl)‐2‐(4‐alkylpiperazin‐1‐yl) acetamide derivatives^22^
	T	CACNA1I	CaV3.3	Brain	Schizophrenia risk gene,^14^ Neurodevelopmental disorders^23^	μ‐Theraphotoxin Pn3a,^24^ TAT‐C3P peptide^25^

The CaV1 Ca^2+^ channel family, also known as LTCC, is distinguished from other VGCCs by their large single‐channel conductance and high sensitivity to organic calcium channel inhibitors and activators. They were named ‘L’ in early studies because of their long‐lasting inward currents during depolarization, which allowed them to be distinguished from rapidly decaying calcium currents, termed transient or T‐type channels.^8^ The LTCCs family members can be categorized into four subtypes according to the corresponding genes encoding the α1 subunit, which are CaV1.1 to CaV1.4. The expression pattern of CaV1.1 and CaV1.4 channels is highly restricted, with CaV1.1 expression mainly limited to skeletal muscle and CaV1.4 expression mainly limited to the retina and immune cells.^8^, ^13^ CaV1.2 and CaV1.3 are widely expressed in essentially all electrically excited cells (including heart, smooth muscle, pancreas, adrenal glands and especially brain), and CaV1.2 and CaV1.3 are usually present in the same cell.^26^ The α1 subunits of CaV1.2 and CaV1.3 have a high degree of sequence homology in the voltage‐sensitive and pore‐forming regions, and despite their close structural relationship, they differ in their biophysical properties as well as their relative abundance in many cells, suggesting that they are capable of performing different physiological functions. It was found that CaV1.3 channel can be activated at more negative voltages than CaV1.2 channel,^27^ and that the CaV1.3 underlies the ‘low‐voltage‐activated’ L‐type currents observed in various tissues.^28^, ^29^

CaV1.3 is encoded by the CACNA1D gene. There is mounting evidence indicating that CaV1.3 plays a significant role in regulating gene expression, cellular motility, cell division, apoptosis, tumorigenesis and metastasis. Additionally, it is involved in facilitating the influx of calcium ions into excitable cells and various calcium‐dependent processes such as muscle contraction and the release of hormones or neurotransmitters. CaV1.3 knockout (CaV1.3^−/−^) mice and humans with mutations in the CACNA1D gene resulting in inactivation of CaV1.3 channels are commonly used paradigms for studying the physiological role of CaV1.3 channels. The tight coupling of CaV1.3 calcium inward flow and synaptic vesicle release in cochlear inner hair cells underlies the formation of hearing.^30^ In the heart, CaV1.3 channels dominate in the sinoatrial node (SAN) and atrioventricular node where CaV1.3 Ca^2+^ influx at negative potentials drives the diastolic depolarization required for normal cardiac pacemaking.^28^ Similar to CACNA1D knockout mice, human CACNA1D mutations cause clinical manifestations of deafness and bradycardia, a condition known as SANDD syndrome.^31^, ^32^ CaV1.3 is expressed in retinal neurons, and although there are no reports of significant visual defects in animals or humans carrying CaV1.3 mutations, a growing body of researches point to its important role in vision formation.^9^, ^33^, ^34^, ^35^ CACNA1D mutations are also associated with aldosterone‐producing adenomas.^36^, ^37^, ^38^

Because of the high expression of CaV1.3 in the brain, CaV1.3 is strongly associated with neuropsychiatric disorders. Blocking CaV1.3 channel in adult substantia nigra pars compacta dopaminergic (SNcDA) neurons induces restoration of the juvenile pacing form, which can protect vulnerable SNcDA neurons and is a novel strategy that can slow or halt Parkinson's progression.^39^ In addition, levodopa‐induced dyskinesia (LID) is an unresolved challenge in Parkinson's treatment. Striatal CaV1.3 gene silencing prevents and ameliorates levodopa dyskinesia, providing new possibilities for CaV1.3 gene therapy for LID.^40^, ^41^ CACNA1D is a gene associated with neurodevelopmental disorders,^42^ and its missense mutations cause diverse and complex disease symptoms.^29^, ^30^, ^31^, ^32^, ^33^ CaV1.3 is also associated with hippocampus‐related cognitive functions^43^ and consolidation of conditioned fear.^44^ In addition, CaV1.3 channel plays an important role in addictive behaviours^45^ and mood disorders,^46^, ^47^ and is potential therapeutic target for psychiatric disorders such as bipolar disorder and major depressive disorder.^33^, ^48^ CaV1.3 is localized in the kidney and mediate Ca^2+^ influx in the proximal tubule with Na/H exchanger isoform 8 jointly.^10^ CaV1.3 is important in human glucose‐induced insulin secretion and common variants of CACNA1D may be associated with type 2 diabetes mellitus.^49^ CaV1.3 is also involved in intestinal Ca^2+^ absorption.^50^, ^51^

## 3 CaV1.3 IN DIFFERENT CANCERS

**FIGURE 2:**
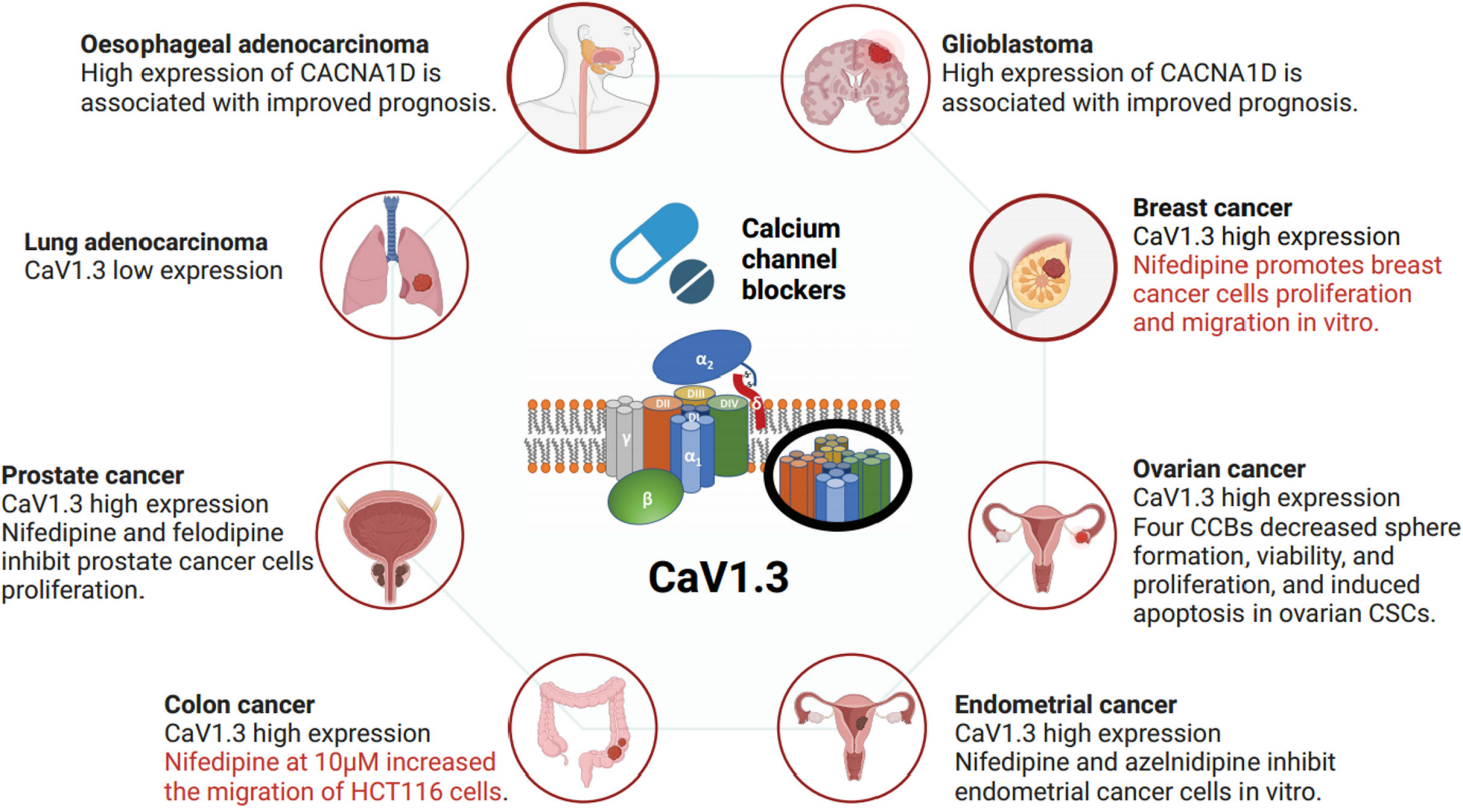
Expression of CaV1.3 in several common cancers and the role of CCBs in cancer cells (Created in BioRender.com).

Despite the growing scientific interest in the role of calcium channels in cancer, the mechanism of calcium channel action in cancer biology remains unelucidated. Calcium channels are intimately involved in complex mechanisms related to calcium homeostasis and contribute to tumour proliferation, invasion and metastasis or drug resistance, but there is no evidence that calcium channels initiate tumour transformation of cells. Thus, whether the increased upper‐regulated calcium channel regulates the oncogenesis processes or invasion capacity by altering the Ca^2+^ signature both sides of the membrane system and membrane voltage, the pH value or other indicators initiatively, or act as a regulatory factor to maintain calcium homeostasis passively still remains unclear. Huber^52^ first came up with a concept to describe the ion channels which experimental interference with their function often impairs cancer cell growth or survival as ‘oncochannels’. CaV1.3 is the most commonly altered ion channel in cancers and CACNA1D shows high expression in most types of cancer (Figure 2).^53^ CaV1.3 is more likely to act as a prototype of the oncochannel in tumour oncogenesis. Targeting CaV1.3 for cancer therapy has attracted great interest among oncologists, and several studies have demonstrated its feasibility in cell lines and animal models. The expression of CaV1.3 correlates with patient prognosis, and mutations in the CACNA1D gene in cancers are becoming clearer, so that CaV1.3 may also be used as a potential marker for determining risk of disease, prognosis, and so on. We also observed that the majority of CaV1.3‐related cancers were also sex hormone‐related (especially prostate, endometrial and breast cancers), and that the sex hormone‐CaV1.3 axis might be contributing significantly to tumorigenesis and development.

### 3.1 Prostate cancer (PCa)

Epidemiological investigations have shown a reduced incidence of PCa^54^ in users of L‐type calcium channel blockers (CCBs) compared to the general population.^55^, ^56^ CACNA1D gene is highly expressed in PCa at both mRNA and protein levels,^57^ and overexpression of the CACNA1D gene is associated with castration‐resistant^57^ and aggressive phenotype.^58^, ^59^ Blockade of L‐type channel's function or down‐regulation of CACNA1D gene expression significantly inhibited androgen‐stimulated intracellular calcium ion efflux, androgen receptor (AR) trans‐activation and cell growth in PCa cells.^57^ Addition of nifedipine and tetrandrine was observed to significantly depress cell proliferation of PCa cell and related AR mediated gene.^60^ Besides, knockout of CACNA1D with siRNA can disable transcription of AR.^60^

Fusions of the male regulatory gene TMPRSS2 and the oncogene ERG (TMPRSS2: ERG or T2E) are common in PCa, and PCa containing gene fusions are thought to represent a distinct disease subtype. Several research groups have identified the CACNA1D gene as a downstream target of the TMPRSS2‐ERG fusion gene in PCa cells.^61^, ^62^, ^63^ The expression level of CACNA1D in the TMPRSS2‐ERG fusion‐positive group was significantly higher than that in the negative group.^57^ Geybels et al. examined the association of CCB use with PCa risk and PCa molecular subtypes defined by T2E status and found that CCB use (ever vs. never) was not associated with overall PCa risk, but the use of CCBs was associated with a reduced Gleason score and relative risk of T2E‐positive PCa.^64^

Androgen deprivation therapy (ADT) is the mainstay of treatment for advanced PCa, but almost all patients progress to castration‐resistant prostate cancer (CRPC) after the initial response to treatment. Bioinformatics analysis revealed elevated CACNA1D gene expression in ADT‐treated PCa patients.^65^ This was confirmed in LNCaP xenograft mice in vivo and in an in vitro PCa cell line model, with a significant increase in CaV1.3 protein expression after bicalutamide ADT.^65^ Under ADT, specific CaV1.3 isoforms (a shortened 170 KD CaV1.3 isoform associated with plasma and cell membranes fails to induce calcium inward flow after membrane depolarization) mediate elevation of basal cytoplasmic calcium and upregulation of store‐operated calcium entry, a mechanism that promotes proliferation and survival of ADT‐resistant CRPC cells.^65^

### 3.2 Endometrial cancer (EC)

Our previous study showed that increased serum ionized calcium promotes peritoneal metastasis and lymph node metastasis in EC,^66^, ^67^ and high levels of serum ionized calcium were significantly associated with advanced EC progression.^68^ We found that CaV1.3 was overexpressed in atypical hyperplasia and EC tissues and elucidated that 17β‐estradiol (E2) and β‐estradiol‐6‐(O‐carboxymethyl) oxime: bovine serum albumin (E2‐BSA) upregulated CaV1.3 expression in EC cell.^69^ The role of E2 in EC development and progression is self‐evident. E2 binds with nuclear oestrogen receptor (ER), regulates the transcription of specific target genes.^70^, ^71^ In addition, E2 can cause an increase in intracellular calcium through a membrane‐triggered effect and activate a kinase cascade reaction in normal and cancerous epithelial cells within minutes.^72^ This process, the so called ‘non‐gene‐transcription effect’, differentiates with regular gene transcription and protein synthesis, acting as an important role in the signal regulation.^69^, ^73^ CaV1.3 was activated by G protein‐coupled oestrogen receptor (GPER/GPR30) on the cell membrane surface regulated with E2, inducing Ca^2+^ influx and activating the downstream signal transduction pathway (ERK1/2/CREB) rapidly, in that promoting the proliferation and migration of EC.^69^ E2‐induced extracellular calcium influx also promotes EC progression by modulating lysosomal activity and mitochondrial reactive oxygen species (ROS).^74^ We also observed autophagy inhibition by 3‐methyladenine leads down‐regulation of CaV1.3 and enhances nifedipine induced cell death.^75^ CCBs have a very promising future in the treatment of EC. We screened azelnidipine (AZL) from 19 FDA‐approved CCBs and found that AZL was the most effective in inhibiting the proliferation of multiple EC cell lines as well as four advanced patient‐derived cells.^76^ However, Ruan et al. detected CaV1.3 mRNA in mouse endometrial epithelial cells (EECs) and their patch‐clamp studies showed that nifedipine‐sensitive and voltage‐dependent inward whole‐cell currents exhibited in mouse EECs.^77^ CaV1.3 may be involved in embryo implantation and interventions against it may affect fertility in patients with EC with preserved reproductive function, so caution should be exercised in this population.

### 3.3 Breast cancer

CaV1.3 is highly expressed in breast cancer and is associated with poor patient prognosis.^78^, ^79^ Similar to our study in EC,^69^ E2 upregulates CaV1.3 expression via GPR30 in MCF‐7 cells in dosage and time‐dependent manner.^78^ Moreover, E2 induced Ca^2+^ influx through CaV1.3 to activate the expression of p‐ERK1/2 for cell proliferation.^78^ Importantly, ultrasound‐targeted microbubble disruption of CaV1.3 siRNA used in breast cancer xenograft mice significantly inhibits tumour growth and improves survival.^78^ Which goes beyond conventional CCBs and provides new ideas and tools for targeting CaV1.3 to treat cancer. Jacquemet et al.^79^ found that endogenous CaV1.3 α1 subunit and MYO10 are localized at the tip of the filopodia and that silencing of CACNA1D gene expression by siRNA oligonucleotides reduces the formation of filopodia and invasion of breast cancer cells. CaV1.3 may direct cell migration and promote cancer cell invasion by affecting filopodia stability. Intriguingly, Guo et al.^80^ reported that nifedipine, but not verapamil, promotes the proliferation and migration of the breast cancer cell line MDA‐MB‐231 in vivo and in vitro via the axis of miRNA‐524‐5p‐BRI3–Erk pathway rather than by regulating [Ca^2+^]_i_. Another study also showed that nifedipine stimulates the proliferation and migration of different breast cancer cells through different pathways (the effect on MCF‐7 cells was through the Akt‐eNOS‐NO axis, whereas the effect on MDA‐MB‐231 cells was through the activation of the ERK axis).^81^ Therefore, nifedipine, a common medication used in the treatment of hypertension, may not be the best choice of medication for breast cancer patients. In addition, a retrospective study of the use of nifedipine as an antihypertensive drug after a diagnosis of breast cancer is overdue. Extrachromosomal circular DNAs (eccDNAs), markers of genomic instability, interact extensively with chromatin and eccDNAs, which allows eccDNAs to act as mobile transcriptional enhancers to promote cancer progression.^82^ Circular amplification of the CACNA1D gene may be an important event in the tumorigenesis and progression of breast invasive carcinoma.^83^

### 3.4 Colon cancer

Colorectum cancer (CRC) also shows CaV1.3 was up‐regulated that only α1D was found overexpressed in CRC tissues compared to healthy tissues among all α1 proteins.^84^ Immunohistochemistry assays revealed the α1D staining was stronger in adenoma and adenocarcinoma tissues compared to normal tissues and in adenoma tissues compared to adenocarcinoma tissues.^84^ In the HCT116 colon cancer cell, the α1D protein of CaV1.3 appears to be predominantly distributed in the cytoplasm and nucleus, with less distribution in the plasma membrane; and lacks a detectable voltage‐gated inward Ca^2+^ current.^84^ α1D protein can regulate basal [Ca^2+^]_c_ either by activating a constitutive Ca^2+^ entry of Ca^2+^ from extracellular side or by promoting Ca^2+^ release from intracellular stores such as the endoplasmic reticulum.^84^ Fourbon et al. showed that α1D in colon cancer is involved in the regulation of Ca^2+^ homeostasis and cell migration through a mechanism that is independent of its plasma membrane classical function but involves the plasma membrane Na^+^/Ca^2+^ exchanger1/3(NCX1/3).^84^

### 3.5 Other cancers

Upregulation of CACNAD1 gene expression was also found in superficial bladder cancer.^85^ In contrast, CACNA1D was significantly down‐regulated in lung adenocarcinoma tissues compared to normal tissues and may be a key ion channel gene altered during tumorigenesis and progression.^86^, ^87^ The Lewis lung carcinoma (LLC) model is the only reproducible syngeneic murine model for lung cancer, and whole‐exome sequencing (WES) performed on the LLC cell line identified a deleterious mutation in CACNA1D.^88^ A double‐hit event in the CACNA1D gene was observed in a study of WES of hereditary diffuse gastric cancer (HDGC), which may be an important mechanism for HDGC tumorigenesis.^89^ CACNA1D‐ERC2 is a newly identified potential oncogenic fusion gene in malignant pleural mesothelioma (MPM) and may be an early event in MPM evolution.^90^ WES reveals that mutations in CACNA1D are also present in urothelial clear cell carcinoma.^91^ CACNA1D is important for maintaining the stemness of ovarian cancer stem cells (CSCs) and is associated with poor prognosis in patients with ovarian cancer.^92^ Four CCBs were screened to reduce ovarian CSCs sphere formation, viability and proliferation, and to induce apoptosis (associated with inhibition of AKT and ERK signalling).^92^ Importantly, the combined use of manidipine and paclitaxel showed enhanced effects in an ovarian CSC xenograft mouse model.^92^ Therefore, CACNA1D has the potential to become a biomarker and therapeutic target for recurrence and metastasis in patients with advanced ovarian cancer. In oesophageal adenocarcinoma^93^ and glioblastoma,^94^ High expression of the CACNA1D gene is associated with improved patient prognosis. Therefore, CACNA1D is a potential prognostic marker for some cancers.

## 4 CaV1.3 REGULATION IN CANCER

Although the mechanisms of CACNA1D in regulating cancer metastasis was not completely elucidated, general views illustrate that calcium channels in degradation of the extracellular matrix and the cellular migration, play a pivotal role in this process.^95^ Indeed, VGCC family in cancer has both canonical and non‐canonical functions in regulation of intracellular calcium concentration and are involved in transcriptional regulation of the expression of other proteins including potassium channels.^96^ Thus non‐genomic signalling in CaV1.3 proteolytic cleavage of the c‐terminus that translocated to the nucleus and regulates genes transcription involved in tumour development and progression. Ling Lu et al.^97^ revealed the CACNA1D protein control the activity of the Ca^2+^‐activated K^+^ channel, and the C terminus of CACNA1D translocated to the nucleus and function as a transcription factor to regulate the expression of SK2 channels. Changing the distal C‐terminal end of CACNA1D will alter the properties of its sensitivity of Ca^2+^ blocker dihydropyridines (DHPs) like CaV1.2, disruption of calmodulin‐IQ domain binding in the CaV1.3_Δ41_ and full‐length CaV1.3_42_ channels was related to sensitivity of DHPs.^98^ This raises the intriguing possibility that CCBs of CaVs may be beneficial in cancer treatment α1D protein regulated cytosolic Ca^2+^ concentration by inhibiting NCX.^84^

Currently, AR is dominant factor for PCa development and progression and that reactivation of the AR as a transcription factor is essential in castration‐resistant progression. Previous studies recognized that androgens stimulate Ca^2+^ influx via LTCC in PCa cells,^99^ androgens can induce rapid Ca^2+^ flux in a variety of cell types,^100^ but the responsible subtype of calcium channel is not yet identified. Evidence proved silencing AR expression with an adeno‐associated virus–delivered AR silencing construct will eliminate R1881‐stimulated Ca^2+^ influx, which demonstrated that androgen stimulated Ca^2+^ influx involves CaV1.3 calcium channel is rely on AR.^57^ E2 can also modulate CaV1.3 through the oestrogen receptor, as we described earlier.

In most solid tumours, hypoxia is a feature of the tumour microenvironment.^101^ Hypoxia‐inducible factor‐1 (HIF‐1) is a transcription factor widely recognized as a major regulator of the transcriptional response to hypoxia, orchestrating cellular adaptive mechanisms triggered in response to hypoxic environments. LTCCs show a particularly high sensitivity to hypoxia compared to other channel types.^102^ CaV1.3 in neuroblastoma cells is downregulated by hypoxia, and the mechanism of this downregulation may be through the HIF‐1 pathway.^103^ Hypoxia increases Ca^2+^ concentration and induces HIF‐1α protein expression in PC12 cells (derived from rat adrenal pheochromocytoma).^104^ LTCCs Cav1.2 and CaV1.3 are also upregulated by hypoxia.^104^ However, echinocandins (a HIF‐1α inhibitor) only slightly and dose‐dependently decreased the expression of the CaV1.2 gene but not the Cav1.3 gene.^104^ CaV1.2 but not Cav1.3 may be a downstream target gene of HIF‐1α involved in the regulation of PC12 cell death under hypoxic conditions.

**FIGURE 3:**
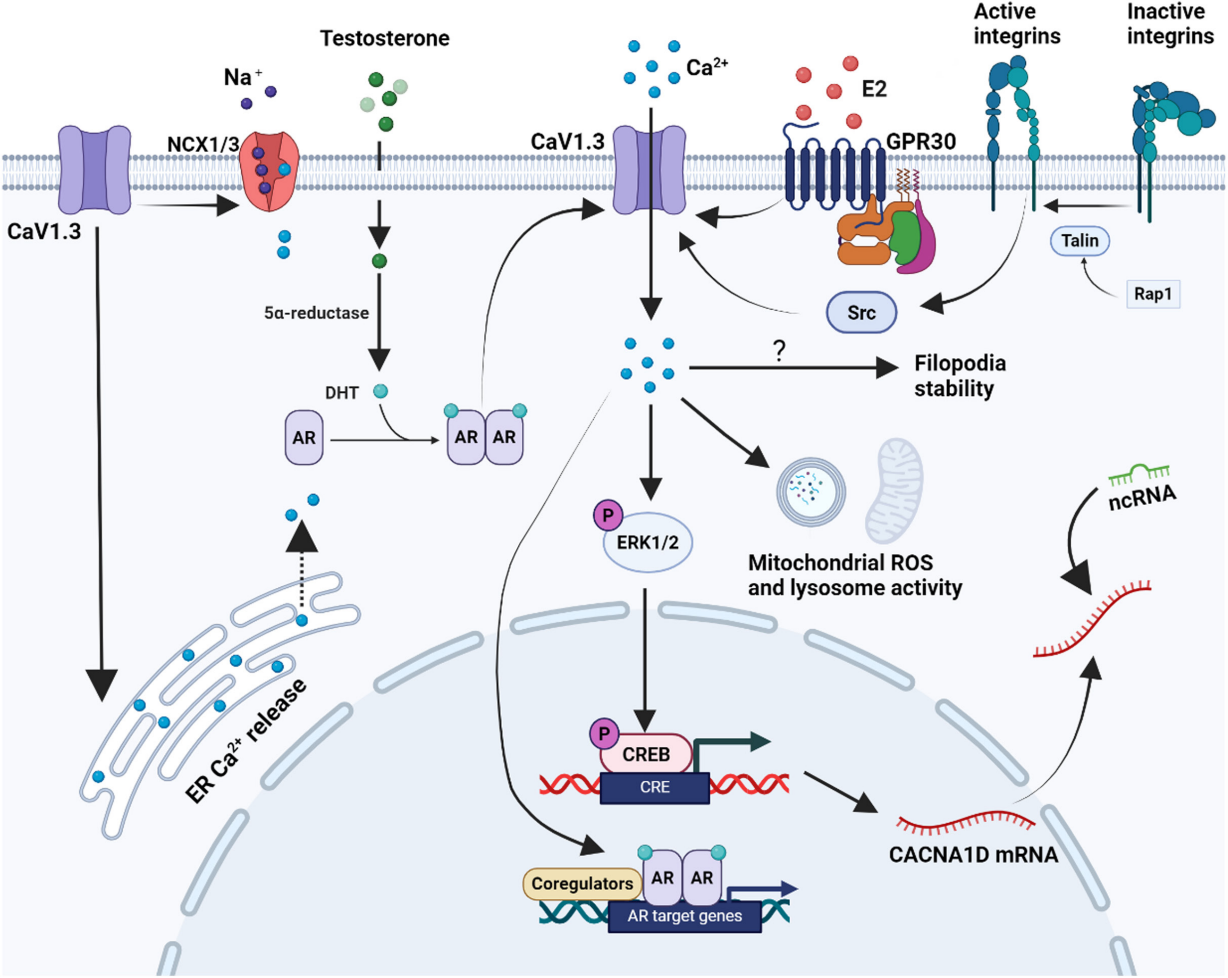
Schematic representation of the regulation of CaV1.3 in cancers. In prostate cancer cells (LNCaP cells), androgen‐stimulated Ca^2+^ influx involves CaV1.3 calcium channels, which are also AR‐dependent.^57^ L‐type channel Cav1.3‐dependent Ca^2+^ influx is essential for AR‐mediated gene expression.^57^ In endometrial and breast cancers, oestrogen mediates CaV1.3 calcium influx via GPR30, activates p‐ERK1/2 expression, and enhances CaV1.3 expression.^69^, ^78^ In EC, oestrogen‐induced extracellular Ca^2+^ influx promotes EC progression by regulating lysosomal activity and mitochondrial ROS.^74^ In breast cancer, integrin inside‐out signalling promotes Ca^2+^ entry at filopodia tips, via CaV1.3, and subsequent filopodia stability.^79^ In colon cancer, α1D protein regulates the migration and invasion of HCT116 cells and its intracellular Ca^2+^ concentration by a mechanism that did not depend on its plasma membrane canonical function but that involved plasma membrane NCX1/3 exchangers and ER Ca^2+^ release.^84^ CACNA1D may also be regulated by some non‐coding RNAs (e.g. TRF‐Val‐CAC‐016 and miR‐371a‐3p). DHT, Dihydrotestosterone; E2: 17β‐estradiol; AR, Androgen receptor; ER, Endoplasmic reticulum; NCX1/3, Na^+^/Ca^2+^ exchanger 1/3; GPR30, G protein‐coupled oestrogen receptor; CREB, CAMP response element binding protein; CRE, CAMP response element; ncRNA, Non‐coding RNA. (Created with BioRender.com).

As the ‘dark matte’ of the genome, non‐coding RNAs have attracted much attention in recent years, and some studies suggest that non‐coding RNA molecules may regulate CACNA1D and its downstream signalling pathways to affect cancers.^105^ TRF‐Val‐CAC‐016, a tsRNA molecule with significantly down‐regulated expression in gastric cancer tissues, inhibits the proliferation of gastric cancer cells by regulating the CACNA1D‐mediated MAPK signalling pathway.^105^ Also, based on mRNA and miRNA expression profiles, Zhang et al.^106^ illustrated the molecular mechanism of PCa, and shows CACNA1D was significantly dysregulated and can be regulated by miR‐371a‐3p. Based on the available studies we mapped the regulation of the CACNA1D gene (Figure 3).

## 5 PHARMACOLOGICAL INSTRUMENTS OF CaV1.3

**FIGURE 4:**
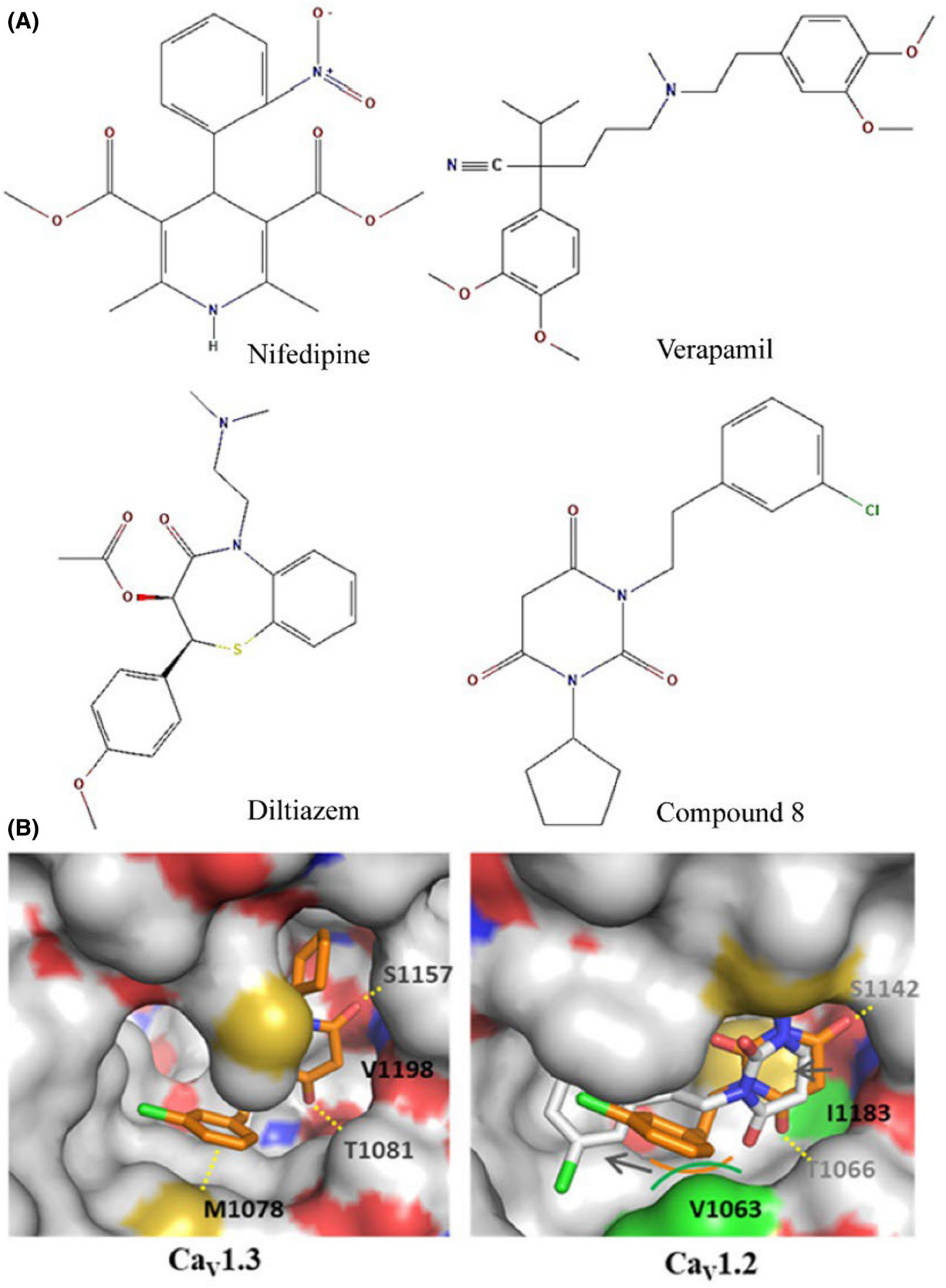
The structural formula of the LTCC blockers, and PYT's mechanism for CaV1.3 preferences. (A) Common blockers of L‐type calcium channels (nifedipine, verapamil, and diltiazem, which are used in the treatment of hypertension) and compound 8 (which may be selective for CaV1.3). (B) Targeted mutagenesis based on homology modelling of the CaV1.3 and CaV1.2 channels revealed that a single amino acid residue within the DHP‐binding pocket (M1078) resulted in a higher selectivity of PYT for CaV1.3 than for CaV1.2. This graph is quoted from Cooper G et al.^131^

L‐type CCBs include DHPs, diltiazem, and phenylalkylamines (Figure 4A). CaV1 channels are sensitive to the dihydropyridine class of CCBs, which includes nifedipine, nimodipine, nisoldipine, felodipine and isradipine and can be activated by DHPs such as Bay K8644. These drugs, rather than physically blocking the pore, act allosterically to shift the channel toward the open or closed state.^107^ Within the CaV1 and CaV3 subfamilies, there is some differential sensitivity where CaV1.2 is more sensitive to nifedipine than CaV1.3 which is incompletely inhibited; moreover, CaV3 channels are relatively less sensitive.^108^ Phenylalkylamines, for example, verapamil, are intracellular pore blockers, which are thought to enter the pore from the cytoplasmic side of the channel and cause occlusion.^109^ CaV1 channels are also sensitive to the CCB family of benzothiazepines such as diltiazem whereas CaV2 and CaV3 are not affected. In mammalian cells, the intracellular and extracellular Ca^2+^ homeostasis levels are illustrated to be crucial for cell progression and proliferation. The transients of Ca^2+^ are also demanding during early G1 and the G1/S phase as well as several stages of mitosis, particularly at the metaphase/anaphase transition and during cytokinesis.^110^, ^111^, ^112^, ^113^, ^114^ Though the relationship between CCBs and cancer still remains controversial in previously studies.^115^, ^116^ The role of T‐type Ca^2+^ channels in cancer cells and the calcium channels blockers as anticancer agents have been discussed in these excellent reviews.^117^, ^118^, ^119^ Besides, several studies have observed negative correlation between CCBs with the PCa risk,^56^, ^120^ or largely unrelated to the risk of invasive breast cancer among women.^121^, ^122^ CaV1.3 plays an important role in the proliferation and survival of cancer cells, but there are still no available specific blockers in experimental studies and clinical applications. For the reason of CaV1.3 compared with the close analogues of VGCCs family (especially CaV1.2), neither current antagonist and agonist works well on the specificity due to their high sequence homology, and the blockers have similar properties in pharmacological, and high doses classic CCBs has non‐selective effects of these subtypes, including DHPs, phenylalkylamines and benzothiazepines. Common inhibitors have been mentioned used in cancer therapy for signal transduction.^123^

CaV1.2 and CaV1.3 underlie the major L‐type Ca^2+^ currents in the mammalian central nervous system. Owing to their high sequence homology, the two channel subtypes share similar pharmacological properties, and at high doses classic CCBs, such as DHPs, phenylalkylamines and benzothiazepines, do not discriminate between the two channel subtypes. Among the existing drugs, DHPs are the best characterized with respect to their potential selectivity for CaV1.3. Independent studies have found that DHPs such as nifedipine,^124^ nimodipine,^98^ and isradipine^125^ inhibit heterologously expressed CaV1.3 channel with about 5–10 folds higher IC50‐values than CaV1.2 under the same experimental protocol. Professor Richard B. Silverman's group synthesized more than 100 modified DHPs^126^ and a variety of hydropyrimidines^127^ in an attempt to obtain highly selective CaV1.3 antagonists, but the best of these compounds were no more than two to three times as selective for CaV1.3 as CaV1.2. This suggests that the binding sites of DHPs are very similar in CaV1.3 and CaV1.2.

In 2012, Kang et al.^128^ first reported pyrimidine‐2,4,6‐triones (PYT) was used as a potential scaffold, and the structure–activity relationship‐based modification of this scaffold led to 1‐(3‐chlorophenethyl)‐3‐cyclopentylpyrimidine‐2,4,6‐(1H, 3H, 5H)‐trione (also referred to as compound 8, Figure 4A), a potent and highly selective CaV1.3 L‐type calcium channel antagonist. It showed >600‐folds more potently selectivity for CaV1.3 than CaV1.2 in a fluorescent imaging plate reader assay. And the pharmacological action was confirmed by whole‐cell patch‐clamp electrophysiology. The highly selective compound 8 points to a novel target therapy for Alzheimer disease and undergoing preclinical evaluation^128^ but has not been commercial prepared. Unfortunately, these findings could not be reproduced by two other groups. Ortner et al.^129^ were unable to observe CaV1.3 selectivity using whole‐cell membrane clamp recordings with a similar pulse scheme and assay conditions as the original paper. Compound 8 was found to reproducibly increase Ca^2+^ inward currents (I_Ca_) in CaV1.3 and CaV1.2 channels expressed in TSA‐201 cells by slowing down the activation, inactivation, and enhancement of tail currents.^129^ And similar effects were observed in native CaV1.3 and CaV1.2 channels in mouse chromatin cells.^129^ The modulation of I_Ca_ by compound 8 is very similar to the action of the LTCC activator FPL64176, and compound 8 may act as an activator rather than a blocker of LTCC currents.^129^ Another study of this compound reported that this compound has its selectivity toward CaV1.3 related to CaV1.2_B15_ channels, which is greatly influenced by the β‐subunit type with the association of its splice isoform variants β1, β2a, β3 or β4‐subunit, especially for β2a‐subunit variant and revealed compound 8 inhibited CaV1.2 more than either CaV1.3_42_ or CaV1.3_42a_ channels.^130^ It can be inferred that the selective inhibition of CaV1.3 by compound 8 is not significant and is highly dependent on the composition of the CaV1.3 splice variant in a particular type of β‐subunit. The selectivity of compound 8 for CaV1.3 continued to be confirmed in studies published in 2020, with 100 μM of the compound inhibiting CaV1.3 and CaV1.2 by 60% and 20%, respectively.^131^ More importantly, a single amino acid residue within the DHP binding pocket (M1078) was found to cause PYT to be more selective for CaV1.3 than CaV1.2 (Figure 4B).^131^ A structure–activity relationship study of N‐(3‐chlorophenethyl)‐N′‐cyclopentylpyrimidine‐2,4,6‐(1H, 3H, 5H)‐trione highlights the importance of PYT scaffold lead optimization, and modifications to PYT may dramatically increase selectivity and binding affinity for CaV1.3.^132^

In recent years studies have reported the potential of compounds or proteins from natural plants and animals as selective inhibitors of CaV1.3. Sclareol, a natural compound derived from the clary sage, has previously been shown to inhibit growth and cell cycle progression in human leukaemia cells.^133^ A recent cell‐based high‐throughput screening assay found that sclareol inhibited CaV1.3 more strongly than CaV1.2 (about two‐folds), but this small difference could not be considered selective for CaV1.3.^134^ Chondrosin is a new cytotoxic protein identified by Scarfì et al. from C. reniformis with a high degree of structural homology to the N‐terminal region of the ryanodine receptor/channel.^135^ Cytotoxicity assays show that chondrosin has selective activity against specific cancer cell lines (RAW 264.7 and L929) and that the toxic effects are mediated through extracellular calcium uptake and intracytoplasmic ROS overproduction.^135^ There are physical and functional interactions between CaV1.3 and ryanodine receptor type 2 that result in extracellular calcium entry.^136^ Since chondrosin is highly similar to ryanodine receptors, chondrosin may interact specifically with Ca^2+^ channels expressed on target cell membranes. The active ingredient of golden‐flowered tea may inhibit non‐small cell lung cancer with epidermal growth factor receptor mutations by targeting CACNA1D, offering new possibilities for the treatment of small cell lung cancer.^137^

In conclusion, the need for CaV1.3 selective inhibitors is undoubtedly urgent, both for experimental tools and for cancer therapeutic agents. However, there is no published evidence for an effective CaV1.3‐selective blocker, so the development of highly selective CaV1.3 blockers is still challenging. A recent article provides an excellent overview of the current state of development of CaV1.3‐selective inhibitors and suggests that small molecules are considered to be the basic criterion for CaV1.3 selectivity.^4^

## 6 CONCLUDING REMARKS AND FUTURE PERSPECTIVES

VGCCs has become a pharmacological target for common human diseases by regulating gene expression, cell motility, cell division and cell death processes, maintaining action potential and membrane secretion. CaV1.3, a member of the L‐type calcium channel family, shows high expression in cancer tissues such as PCa and EC, and correlates with poor patient prognosis. The crucial role of CaV1.3 in various cancers is increasingly being comprehended and regarded as a potential target for cancer therapy. Moreover, the search for specific inhibitors of CaV1.3 is currently a great challenge. Currently used DHPs lack selectivity for CaV1.3, and even studies have reported that nifedipine promotes breast cancer proliferation and migration in vitro. The development of selective CaV1.3 blockers is in urgent demand and is key to the translation of basic research to the clinic, and we expect that highly selective CaV1.3 blockers will be available for targeted cancer therapy in the near future.

On the one hand, CaV1.3 is widely distributed in the human body, especially in the nervous system and cardiovascular system and the systemic use of CaV1.3 inhibitors will undoubtedly bring some unwanted effects. On the other hand, the doses of CCBs used to suppress cancer in vitro studies are quite high and cannot be clinically achieved without significant toxicity.^138^ How to target and regulate CaV1.3 in cancer cells to exert anti‐tumour effects is an issue to be considered in the future. In addition, with the rapid development of material science, the materialistic modification of existing CCBs is a new idea to reduce side effects and increase therapeutic efficacy. As in our study, liposome encapsulation of AZL to form nanoparticles (NP@AZL) for drug delivery can kill EC cells more effectively.^76^ A number of CCBs have been used in clinical trials for the treatment of cancers, with safety and efficacy gradually being demonstrated.^139^, ^140^ However, a large number of clinical trials and evidence‐based medicine evidences are needed to confirm the safety and efficacy of L‐type CCBs in the treatment of cancers. Moreover, CaV1.3‐based gene therapy deserves our attention, and studies have confirmed its potential.^78^

In view of the current researches of CaV1.3 in cancers, we have the following thoughts: (i) Calcium homeostasis regulation is a complex process, does CaV1.3 in cancers exist reciprocal regulation with other types of calcium channels and if so, how is it achieved? (ii) What is the mechanism by which CaV1.3 acts as an oncogene in most cancers and cancer suppressor gene in a few? (iii) Current studies seem to focus only on CaV1.3 in the cell membrane and calcium ions in the cytoplasm, while relatively little attention has been paid to calcium ions in the nucleus. CaV1.3 is also distributed in the nucleus, so is it involved in nuclear calcium regulation? (iv) The sex hormone‐CaV1.3 axis appears to play an essential role in tumorigenesis, the details of which need to be further explored.

In conclusion, CaV1.3 is an important potential target for cancer therapy. The development of selective inhibitors of CaV1.3 and the safety of systemic application of CaV1.3 inhibitors are the major difficulties that exist. The application of CaV1.3 inhibitors for targeted therapy of cancers requires a great deal of work to be done, including basic research and rigorous clinical trials.

## AUTHOR CONTRIBUTIONS

**Xuerun Liu:** Conceptualization (equal); formal analysis (lead); investigation (lead); software (equal); writing – original draft (lead). **Boqiang Shen:** Formal analysis (equal); writing – original draft (equal). **Jingyi Zhou:** Writing – review and editing (equal). **Juan Hao:** Methodology (equal); software (equal); writing – original draft (equal). **Jianliu Wang:** Funding acquisition (lead); supervision (lead); writing – review and editing (lead).

## FUNDING INFORMATION

This work was supported by grants from the National Natural Science Foundation of China (No.82230050, 81902635 and 82372621).

## CONFLICT OF INTEREST STATEMENT

The authors confirm that there are no conflicts of interest.

## Data Availability

The data that support the findings of this study are available from the corresponding author upon reasonable request.
